# Development and Validation of a HPLC-MS/MS Method for Simultaneous Determination of Twelve Bioactive Compounds in *Epimedium*: Application to a Pharmacokinetic Study in Rats

**DOI:** 10.3390/molecules23061322

**Published:** 2018-05-31

**Authors:** Mengjie Sun, Yanwei Yin, Juan Wei, Xiaopeng Chen, Huizi Ouyang, Yanxu Chang, Xiumei Gao, Jun He

**Affiliations:** Tianjin State Key Laboratory of Modern Chinese Medicine, Tianjin University of Traditional Chinese Medicine, 312 Anshanxi Road, Nankai District, Tianjin 300193, China; 15122865529@163.com (M.S.); Yinyw8739@163.com (Y.Y.); 15222797261@163.com (J.W.); xpchen@tjutcm.edu.cn (X.C.); huihui851025@163.com (H.O.); tcmcyx@126.com (Y.C.); gaoxiumei@tjutcm.edu.cn (X.G.)

**Keywords:** *Epimedium* extract, pharmacokinetics, HPLC-MS/MS, rat plasma

## Abstract

A rapid and reliable HPLC-MS/MS method has been developed and validated for the simultaneous quantification of twelve bioactive compounds (baohuoside II, baohuoside I, sagittatoside A, sagittatoside B, magnoflorine, epimedin A, epimedin B, epimedin C, chlorogenic acid, neochlorogenic acid, cryptochlorogenic acid and icariin) in rat plasma. The collected plasma samples were prepared by protein precipitate with acetonitrile. The twelve compounds were separated on a CORTECS^®^C18 column (4.6 mm × 150 mm, 2.7 μm) with a gradient mobile phase system of 0.1% (*v*/*v*) formic acid and acetonitrile at a flow rate of 0.3 mL/min. All of the analytes were quantitated using electrospray ionization (ESI) in negative ion mode with selected reaction monitoring (SRM). The intra- and inter-day accuracy ranged from −5.6% to 13.0%, and the precisions of the analytes were less than 10.9%. The mean recoveries of the analytes were in the range of 60.66% to 99.77% and the matrix effect ranged from 93.08% to 119.84%. Stability studies proved that the analytes were stable under the tested conditions, with a relative standard deviation (RSD) lower than 11.7%. The developed method was successfully applied to evaluating the pharmacokinetic study of twelve bioactive compounds after oral administration of *Epimedium* extract in rat.

## 1. Introduction

*Epimedium* is a traditional tonic herb used for “reinforcing the Yang and nourishing the kidney” in China. Four species of *Epimedium*, including *Epimedium koreanum* Nakai., *Epimedium pubescens* Maxim., *Epimedium brevicornu* Maxim. and *Epimedium sagittatum* (Sieb. et Zucc.) Maxim., are recorded in Chinese Pharmacopeia (Edition 2015). Traditionally, *Epimedium* are used to treat impotence, forgetfulness and neurasthenia [1]. Nowadays, pharmacological study has demonstrated that *Epimedium* possesses a variety of activities, such as inhibition of tumor, anti-oxidation and anti-inflammatory [2,3]. Meanwhile, clinical research has suggested the treatment of osteoporosis, diabetes, releasing depression, cardiovascular diseases, chronic nephritis and rheumatoid arthritis with *Epimedium* [4,5,6,7].

Phytochemical investigation has found many kinds of compounds isolated and identified from *Epimedium*, such as flavonoid glycosides, phenylpropanoids, alkaloids, polysaccharides, lignins and sesquiterpenes [8]. The most important active compounds are flavonoid glycosides, including icariin, icariside-II, epimedin A and epimedin B, etc. [9]. The flavonoid glycosides show the activity in treatment of inflammatory, metabolic disorder, osteoporosis, diabetes and cancer [10,11,12,13]. Phenylpropanoids including chlorogenic acid, neochlorogenic acid and cryptochlorogenic acid have anti-inflammatory, anti-oxidation and anti-cancer effects [14]. Magnoflorine, the alkaloid from *Epimedium*, displays hypoglycemic activity [15]. In addition, polysaccharides and their chemical derivatives from *Epimedium*, such as glucose, rhamnose and mannose, possess immunomodulatory, anti-tumor, anti-oxidation, and anti-microbial activities [16,17].

Pharmacokinetic study plays an important role in clinical applications with regard to determination of the clinical dose and avoiding side effects. Several manuscripts focusing on the pharmacokinetics of *Epimedium* have been published, such as the validation of an LC–MS/MS method to quantify five flavonoid glycosides (icariin, icaritin, desmethylicaritin, icariside I and icariside II) simultaneously in *Epimedium* [18]. Furthermore, the pharmacokinetic properties of icariin, epimedin A, epimedin B, epimedin C, baohuoside I, sagittatoside B and 2″-*O*-rhamnosyl icariside II have been determined in dogs in one injection [19].

In this study, a reliable HPLC-MS/MS method was developed to quantify twelve bioactive compounds (baohuoside I, baohuoside II, sagittatoside A, sagittatoside B, magnoflorine, epimedin A, epimedin B, epimedin C, chlorogenic acid, neochlorogenic acid, cryptochlorogenic acid and icariin) simultaneously in rat plasma. Then the assay was applied to the pharmacokinetic study of the compounds in rats after oral administration of *Epimedium* extract.

## 2. Results and Discussion

### 2.1. Method Development

**Figure 1:**
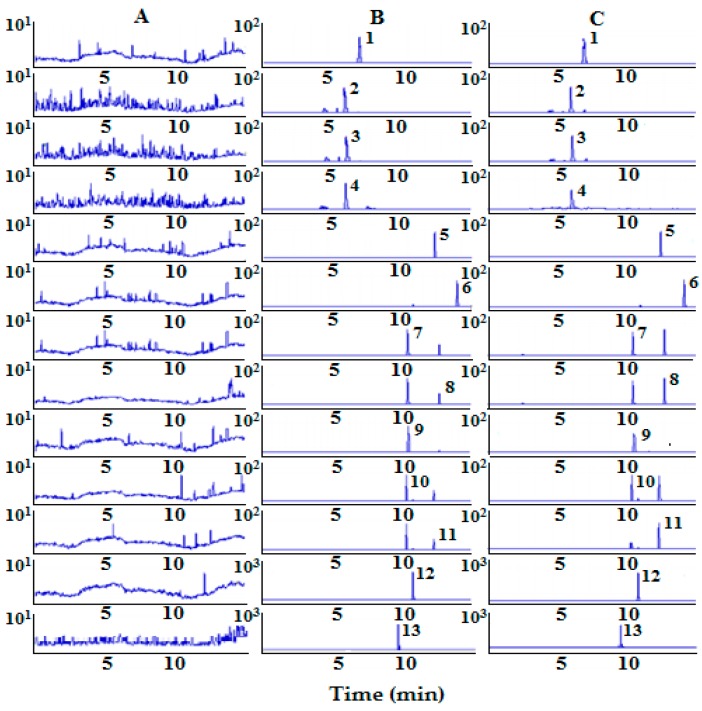
SRM chromatograms of magnoflorine (1), neochlorogenic acid (2), chlorogenic acid (3), cryptochlorogenic acid (4), baohuoside II (5), baohuoside I (6), epimedin B (7), sagittatoside B (8), epimedin C (9), epimedin A (10), sagittatoside A (11), icariin (12) and IS (13). (**A**) Blank plasma; (**B**) blank plasma spiked with the analytes and IS; (**C**) plasma sample 15 min after oral administration of *Epimedium* extract.

To obtain a better separation and a shorter retention time, a variety of mobile phases such as acetonitrile-water, acetonitrile-0.1% (*v*/*v*) formic acid, methanol-water and methanol-0.1% (*v*/*v*) formic acid were tested. Finally, acetonitrile-0.1% (*v/v*) formic acid was used as the optimum mobile phase on CORTECS^®^C18 column. As shown in Figure 1, 12 analytes and IS were eluted for 15 min, and no interfering peaks were observed.

To optimize the mass spectrometry conditions, negative and positive modes were tested. The negative mode showed better intensity for analytes and lower background than the positive mode. In addition, the capillary temperature, auxiliary nitrogen pressure, drying gas flow and ion spray voltage were optimized to obtain the most suitable electrospray ionization parameters as described in Section 3.2.

### 2.2. Sample Preparation

To develop a simple and efficient sample preparation method, liquid-liquid extraction and protein precipitation were tested. Liquid-liquid extraction using ethyl acetate achieved good recovery for flavonoid glycosides, but not for chlorogenic acid, neochlorogenic acid and cryptochlorogenic acid. Protein precipitation with methanol and acetonitrile were compared, and acetonitrile precipitation showed better recovery. Moreover, to obtain optimum extraction, different kinds of acids (formic acid, acetic acid and phosphoric acid) were added to acetonitrile and compared. In consideration of everything, the protein precipitation using acetonitrile containing formic acid was used in the sample preparation for all analytes.

### 2.3. Method Validation

#### 2.3.1. Specificity

Blank plasma samples were prepared as described in Section 3.5. The chromatograms of blank plasma (A), blank plasma samples spiked with analytes and IS (B), and plasma samples collected 15 min after oral administration of *Epimedium* extract (C) are shown in Figure 1. The results show that no interfering peaks were observed in the samples.

#### 2.3.2. Calibration Curves

Calibration curves and LLOQs in rat plasma are listed in Table 1. The regression coefficient (r) was greater than 0.9904 for all calibration curves. This demonstrates that the analytes have good linearity over the linear range. The LLOQs of the 12 analytes were less than 10 ng/mL.

#### 2.3.3. Precision and Accuracy

The precision and accuracy of the 12 analytes is summarized in Table 2. As shown in the table, the intra- and inter-day accuracy (RE) ranged from −5.6 to 12.9%, and the precision (RSD) ranged from 0.4% to 10.9%. The results prove that the developed method is accurate and precise.

#### 2.3.4. Extraction Recovery and Matrix Effect

The extraction recoveries and matrix effect of QC samples at three different concentrations are summarized in Table 3. The extraction recoveries of QC samples were in the range of 60.66% to 99.77%, with RSDs of less than 11.3%. The matrix effect ranged from 93.08% to 119.84%, with RSDs of less than 13.2%. The results show that the extraction recovery and the matrix effect are acceptable.

#### 2.3.5 Stability

The stability of QC samples was summarized in Table 4. The RSD of the replicate QC samples was less than 11.7%. The data demonstrates that the analytes are stable at room temperature for 2 h, in an auto-sampler for 12 h, and for 7 days and through three freeze-thaw cycles at −70 °C.

### 2.4. Application

**Figure 2:**
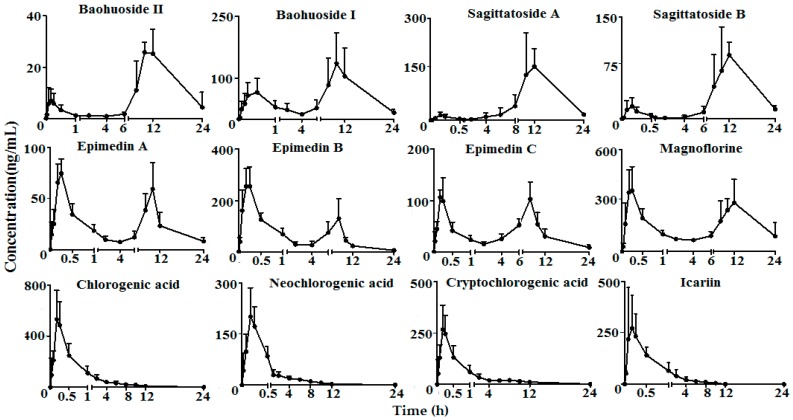
Mean plasma concentration–time curves of baohuoside II, baohuoside 1, sagittatoside A, sagittatoside B, epimedin A, epimedin B, epimedin C, magnoflorine, chlorogenic acid, neochlorogenic acid, cryptochlorogenic acid and icariin in six rats after oral administration of *Epimedium* extract (mean ± SD).

The plasma samples obtained from the rats to which *Epimedium* extract had been administrated by gavage were determined by the HPLC-MS/MS method. Plasma concentration–time curves of twelve compounds in rats are shown in Figure 2, and the major pharmacokinetic parameters are depicted in Table 5. 

According to the pharmacokinetic parameters, the analytes were divided into two groups: one group includes baohuoside I, baohuoside II, sagittatoside A, sagittatoside B, magnoflorine, epimedin A, epimedin B and epimedin C (group A), and the other group includes chlorogenic acid, neochlorogenic acid, cryptochlorogenic acid and icariin (group B). A bimodal phenomenon appeared in the plasma concentration–time curves of the analytes of group A. The *T_max1_* of baohuoside I, baohuoside II, sagittatoside A, sagittatoside B, magnoflorine, epimedin A, epimedin B and epimedin C in group A were 0.38 ± 0.14 h, 0.18 ± 0.03 h, 0.18 ± 0.03 h, 0.20 ± 0.04 h, 0.18 ± 0.03 h, 0.22 ± 0.04 h, 0.19 ± 0.07 h and 0.22 ± 0.04 h, respectively, and the *T_max2_* were 10.00 ± 1.79 h, 10.67 ± 1.03 h, 11.67 ± 0.82 h, 11.00 ± 1.67 h, 10.67 ± 1.63 h, 9.33 ± 0.94 h, 7.67 ± 0.75 h and 7.67 ± 0.75 h, respectively. This bimodal phenomenon of the analytes (epimedin A, epimedin B and baohuoside I) was also found in previous reports [19]. This phenomenon may be caused by glucuronidation or enterohepatic circulation [20]. The *T_max_* of chlorogenic acid, neochlorogenic acid, cryptochlorogenic acid and icariin in group B was 0.19 ± 0.05 h, 0.19 ± 0.05 h, 0.19 ± 0.05 h and 0.16 ± 0.05 h, respectively, which is coincident with the reported literature [21,22]. The *t*_1/2_ of group A and B ranged from 3 h to 18 h and 0.5 h to 1 h, respectively. The results indicated that the analytes of group B were absorbed and eliminated quickly in the rat plasma after oral administration of *Epimedium* extract. Meanwhile, the analytes of group A required a long time to eliminate after absorption.

## 3. Experimental

### 3.1. Chemicals and Reagents

**Figure 3:**
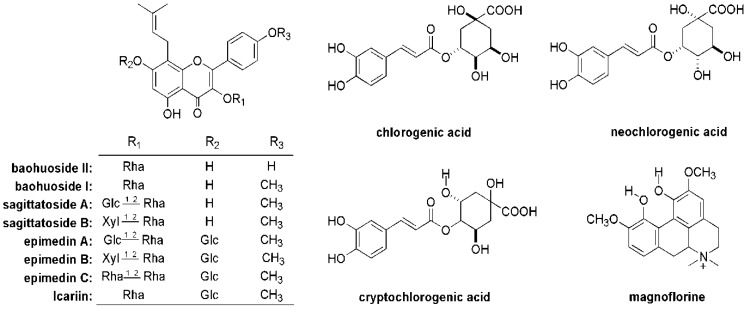
The chemical structures of the 12 compounds.

Methanol and acetonitrile (chromatographic grade) were obtained from Fisher Scientific (Fair Lawn, NJ, USA). The standard compounds of baohuoside I, baohuoside II, sagittatoside A, sagittatoside B, magnoflorine, epimedin A, epimedin B, epimedin C, chlorogenic acid, neochlorogenic acid, cryptochlorogenic acid, icariin and liquiritin (internal standard, IS) (purity ≥ 98%) were provided by YIFANG S&T Co. Ltd. (Tianjin, China). The chemical structures of the compounds are shown in Figure 3. Formic acid (chromatographic grade) was obtained from ROE (St. Louis, MO, USA). Deionized water was supplied by an Alpha-Q water purification system (Bedford, MA, USA).

### 3.2. HPLC-MS/MS Analysis

An Agilent 1200 Series HPLC system, which consisted of a binary pump (G1312A), an auto-sampler (G1367B), an online degasser unit (G1322A), along with temperature-controlled column compartment (G1316A), was used for the analysis. The twelve compounds and IS were separated on a CORTECS^®^C18 column (4.6 mm × 150 mm, 2.7 μm) maintained at 30 °C with the mobile phase consisted of acetonitrile (A) and 0.1% (*v*/*v*) formic acid (B). The flow rate was 0.3 mL/min. The gradient elution was as follows: 0–4 min, 20–50% A; 4–5 min, 50–60% A; 5–15 min, 60–70% A. The injection volume was 5 μL.

The tandem mass spectrometry was performed on a triple-quadrupole mass spectrometer equipped with an electrospray ionization (ESI) source (Agilent G6430A system). Nitrogen gas served as the nebulizer, drying, and collision gas. Analytes were monitored by SRM in negative mode with the electrospray ionization parameters optimized as follows: ion spray voltage of −4000 V, capillary temperature of 350 °C, auxiliary nitrogen pressure of 20 psi, drying gas flow of 9 L/min. The mass spectrometric parameters of precursor ion, product ion, collision energy (CE) and fragmentor (Frag) for analytes are listed in Table 6. Both the collision energy and the fragmentor were optimized for each compound by infusion of the standard solutions of methanol to mass spectrometry directly. The peak in sample was recognized as the target compound if the retention time, precursor ion, and product ion are same as the stand. For each analyte, the most abundant product ion was chosen for quantification. All analytes were quantified using the 6-point calibration curve. The peak areas were used for quantification following an internal algorithm. For each batch of samples processed and analyzed, the determined concentration of each compound in the QC samples, as quantified by the standard curves, was required to fall within ±15% of the known concentration for the data to be included in the final analysis.

### 3.3. Preparation of Extract from Epimedium

For the preparation of *Epimedium* extract, 1000 g of *Epimedium* was extracted twice by refluxing with 16 L of 70% (*v*/*v*) ethanol for 1.5 h each time. The extraction solutions were filtered and combined. Then, the *Epimedium* extract was obtained by evaporating the combined solutions to dryness under reduced pressure. To calculate the dosage of administration, the contents of 12 analytes were determined by the analytical method described in Section 3.2. The contents of baohuoside I, baohuoside II, sagittatoside A, sagittatoside B, magnoflorine, epimedin A, epimedin B, epimedin C, chlorogenic acid, neochlorogenic acid, cryptochlorogenic acid and icariin in *Epimedium* extract were 2.75, 0.61, 0.80, 1.34, 7.32, 3.22, 3.50, 3.54, 3.59, 0.61, 2.14 and 13.81 mg/g, respectively.

### 3.4. Working Solutions

To make the stock solutions, the standards of 12 analytes were separately weighed and dissolved in methanol. The appropriate amount of the 12 stock solutions were mixed and diluted with methanol to get a primary mixed working solution containing 1 μg/mL of baohuoside II; 2 μg/mL of baohuoside I, sagittatoside A, sagittatoside B, epimedin A, epimedin B and epimedin C; 10 μg/mL of chlorogenic acid; 4 μg/mL of magnoflorine, neochlorogenic acid, cryptochlorogenic acid and icariin, respectively. Then, the primary mixed working solution was diluted with methanol to obtain a series of working solutions at appropriate concentrations. Liquiritin was prepared in methanol as IS solution at 1 μg/mL.

The calibration curves were prepared by adding appropriate amounts of the working solutions to blank rats plasma (100 μL) with 20 μL of IS, yielding a series of concentrations at 1, 2, 10, 50, 100, 200 ng/mL for baohuoside II; 2, 4, 20, 100, 200, 400 ng/mL for baohuoside I, sagittatoside A, sagittatoside B, epimedin A, epimedin B and epimedin C; 10, 20, 100, 500, 1000, 2000 ng/mL for chlorogenic acid; 4, 8, 40, 200, 400, 800 ng/mL for magnoflorine, neochlorogenic acid, cryptochlorogenic acid and icariin.

Quality control (QC) samples containing 12 analytes at low, medium and high concentrations of 1, 10, 200 ng/mL for baohuoside II; 2, 20, 400 ng/mL for baohuoside I, sagittatoside A, sagittatoside B, epimedin A, epimedin B and epimedin C; 10, 100, 2000 ng/mL for chlorogenic acid; 4, 40, 800 ng/mL for magnoflorine, neochlorogenic acid, cryptochlorogenic acid and icariin were prepared in the same manner. All the working solutions and QC samples were stored at 4 °C.

### 3.5. Sample Preparation

The plasma sample (100 μL) was mixed with 20 μL of methanol (volume of the corresponding working solution for calibration curve and QC sample), 20 μL of IS (1 μg/mL), 20 μL of formic acid and 400 μL of acetonitrile. Then, the mixture was vortexed for 3 min and centrifuged at 14,000 *g* for 10 min. The upper organic phase (370 μL) was removed to a clean 1.5 mL Eppendorf tube (EP tube) and evaporated to dryness under a gentle nitrogen stream. After that, the residue was dissolved in 100 μL methanol, vortexed for 3 min and centrifuged at 14,000 *g* for another 10 min. Finally, an aliquot of 5 μL of the upper organic layer was injected into the HPLC-MS/MS system for analysis.

### 3.6. Method Validation

#### 3.6.1. Selectivity

The selectivity was carried out by comparing the blank plasma samples from six different rats, blank plasma samples added with analytes and IS, and post-dosing plasma samples to evaluate any potentially interfering substances. 

#### 3.6.2. Calibration Curve

The calibration curves were prepared by assaying standard plasma samples at six concentrations as described in Section 3.4. The linearity of each calibration curve was determined by plotting the ratio of the chromatographic peaks area (analytes/IS) versus the concentration of these analytes with a weighted (1/x^2^) least square linear regression model. Lower limit of quantification (LLOQ) was the lowest concentration of analytes which can achieve a reliable accuracy and precision with signal–noise ratio (S/N) of about 10.

#### 3.6.3. Precision and Accuracy

The intra- and inter-day precision and accuracy were measured by analysis of six replicates QC samples at low, medium and high concentrations (1, 10, 200 ng/mL for baohuoside II; 2, 20, 400 ng/mL for baohuoside I, sagittatoside A, sagittatoside B, epimedin A, epimedin B and epimedin C; 10, 100, 2000 ng/mL for chlorogenic acid; 4, 40, 800 ng/mL for magnoflorine, neochlorogenic acid, cryptochlorogenic acid and icariin) on three consecutive days. Precision was expressed as the RSD, which should not exceed 15% (except for LLOQ less than 20%). The accuracy expressed as the relative error (RE) was assessed by comparing the measured concentration with its true value and accepted within ±15% (except for LLOQ within ±20%).

#### 3.6.4. Extraction Recovery and Matrix Effect

The extraction recovery was determined by comparing the peak areas of six replicates QC samples at three concentrations with post-extraction spiked samples. The matrix effect was assessed by comparing the peak areas of post-extraction spiked samples with working solution at the same concentration on three QC levels.

#### 3.6.5. Stability

The stability of all analytes in rat plasma was investigated by testing QC samples at three concentrations under various conditions as followed: storage at room temperature for 2 h, in auto-sampler for 12 h, at −70 °C for 7 days and three freeze-thaw cycles. The RSDs of the stability should be within 15%.

### 3.7. Pharmacokinetic Studies

Male Sprague-Dawley rats (220 ± 10) g were obtained from the Beijing HUAFUKANG Bioscience Co., Inc. (Beijing, China). Six male rats were acclimatized to the facilities for a week and fasted for 12 h, but allowed free access to water, before dosing. The *Epimedium* extract was suspended in 0.5% carboxymethyl cellulose sodium (CMC-Na) aqueous solution and given to each rat by oral administration at a single dose of 10 g/kg. Blood samples (250 μL) were collected from the fossa orbitalis of rats at 0 (before dosing), 0.03, 0.08, 0.17, 0.25, 0.5, 1, 2, 4, 6, 8, 10, 12 and 24 h after dosing. Then, the blood samples were centrifuged at 6000 *g* for 10 min to obtain the plasma, and the plasma was removed to another EP tube and frozen at −70 °C until analysis. The rat plasma concentration–time data of 12 analytes were computed by the software “Drug and Statistics 1.0” (DAS 1.0)

## 4. Conclusions

In this research, a rapid and reliable HPLC-MS/MS method was established to simultaneously determine twelve bioactive compounds (baohuoside I, baohuoside II, sagittatoside A, sagittatoside B, magnoflorine, epimedin A, epimedin B, epimedin C, chlorogenic acid, neochlorogenic acid, cryptochlorogenic acid and icariin) in rat plasma. The validated method was successfully applied to the pharmacokinetic study of twelve bioactive compounds after oral administration of *Epimedium* extract in rats. Furthermore, this is also the first pharmacokinetic study of baohuoside II and sagittatoside A. The pharmacokinetic study may contribute to the clinical usage of *Epimedium*.

## Figures and Tables

**Table 1:** Calibration curves, correlation coefficients, linear ranges and LLOQ of the analytes.

Compounds	Calibration Curve	r	Linear Range (ng/mL)	LLOQ(ng/mL)
baohuoside II	Y = 1.3944X + 4.6850	0.9930	1.0-200.0	1.0
baohuoside I	Y = 1.4452X + 0.0129	0.9943	2.0-400.0	2.0
sagittatoside A	Y = 0.5707X + 4.8545	0.9918	2.0-400.0	2.0
sagittatoside B	Y = 0.6968X + 7.8080	0.9908	2.0-400.0	2.0
epimedin A	Y = 0.1564X − 8.1949	0.9912	2.0-400.0	2.0
epimedin B	Y = 0.3559X + 5.3440	0.9939	2.0-400.0	2.0
epimedin C	Y = 0.1016X + 6.8756	0.9942	2.0-400.0	2.0
magnoflorine	Y = 0.0260X + 2.4814	0.9928	4.0-800.0	4.0
chlorogenic acid	Y = 0.1274X + 0.0016	0.9922	10.0-2000.0	10.0
neochlorogenic acid	Y = 1.7722 X + 0.0013	0.9956	4.0-800.0	4.0
cryptochlorogenic acid	Y = 0.0187X + 0.0010	0.9904	4.0-800.0	4.0
icariin	Y = 0.1178X + 0.0053	0.9966	4.0-800.0	4.0

**Table 2:** Precision and accuracy of 12 analytes in rat plasma (*n* = 6).

Compounds	Spiked Concentration (ng/mL)	Intra-Day			Inter-Day		
		Measured (ng/mL)	RE (%)	RSD (%)	Measured (ng/mL)	RE (%)	RSD (%)
baohuoside II	1	1.02 ± 0.08	2.0	7.8	1.01 ± 0.08	1.0	7.9
	10	10.18 ± 0.88	1.8	8.6	10.09 ± 0.87	0.9	8.6
	200	197.54 ± 1.41	−1.2	0.7	196.66 ± 3.62	−1.7	1.8
baohuoside I	2	2.03 ± 0.07	1.5	3.4	2.03 ± 0.08	1.5	3.9
	20	19.99 ± 0.98	−0.1	4.9	19.54 ± 0.92	−2.3	4.7
	400	390.27 ± 6.47	−2.4	1.7	391.05 ± 7.15	−2.2	1.8
sagittatoside A	2	2.06 ± 0.10	3.0	4.9	1.98 ± 0.12	−1.0	6.0
	20	20.77 ± 1.01	3.9	4.9	21.00 ± 0.90	5.0	4.3
	400	399.42 ± 5.65	−0.1	1.4	396.13 ± 3.57	−1.0	0.9
sagittatoside B	2	2.08 ± 0.06	4.0	2.9	2.14 ± 0.07	7.0	3.3
	20	20.50 ± 1.36	2.5	6.6	21.07 ± 0.81	5.4	3.8
	400	397.48 ± 7.79	−0.6	2.0	404.06 ± 7.29	1.0	1.8
epimedin A	2	2.13 ± 0.20	6.5	9.4	2.26 ± 0.15	13.0	6.6
	20	19.62 ± 2.14	−1.9	10.9	20.92 ± 1.98	4.6	9.5
	400	397.22 ± 7.08	−0.7	1.8	405.26 ± 5.73	1.3	1.4
epimedin B	2	1.98 ± 0.08	−1.0	4.0	2.00 ± 0.13	0.0	6.5
	20	19.92 ± 1.03	−0.4	5.2	20.87 ± 1.02	4.4	4.9
	400	399.43 ± 3.67	−0.1	0.9	399.44 ± 5.76	−0.1	1.4
epimedin C	2	2.02 ± 0.11	1.0	5.4	2.01 ± 0.07	0.5	3.5
	20	19.90 ± 0.81	−0.5	4.1	19.88 ± 0.89	−0.6	4.5
	400	401.44 ± 3.03	0.4	0.8	400.94 ± 2.37	0.2	0.6
magnoflorine	4	4.05 ± 0.24	1.3	5.9	4.06 ± 0.18	1.5	4.4
	40	41.67 ± 2.42	4.2	5.8	41.10 ± 1.87	2.8	4.5
	800	770.53 ± 16.22	−3.7	2.1	797.84 ± 33.32	−0.3	4.2
chlorogenic acid	10	9.86 ± 0.96	−1.4	9.7	9.89 ± 0.62	−1.1	6.3
	100	94.73 ± 4.35	−5.3	4.6	96.39 ± 6.66	−3.6	6.9
	2000	1888.50 ± 52.19	−5.6	2.8	1987.74 ± 96.63	−0.6	4.9
neochlorogenic acid	4	4.16 ± 0.26	4.0	6.3	4.15 ± 0.19	3.8	4.6
	40	39.69 ± 2.63	−0.8	6.6	40.31 ± 1.47	0.8	3.6
	800	804.76 ± 7.42	0.6	0.9	807.99 ± 10.77	1.0	1.3
cryptochlorogenic acid	4	3.94 ± 0.12	−1.5	3.0	3.92 ± 0.07	−2.0	1.8
	40	40.25 ± 1.39	0.6	3.5	40.63 ± 0.96	1.6	2.4
	800	780.52 ± 16.27	−2.4	2.1	774.79 ± 13.82	−3.2	1.8
icariin	4	4.02 ± 0.15	0.5	3.7	4.02 ± 0.11	0.5	2.7
	40	39.30 ± 1.03	−1.8	2.6	39.85 ± 1.13	−0.4	2.8
	800	801.05 ± 3.35	0.1	0.4	802.21 ± 4.66	0.3	0.6

**Table 3:** Extraction recoveries and matrix effects of the analytes (*n* = 6).

Compounds	Spiked Concentration (ng/mL)	Extraction Recovery (%)	RSD (%)	Matrix Effect (%)	RSD (%)
baohuoside II	1	67.78 ± 1.75	2.6	119.03 ± 3.54	3.0
	10	84.44 ± 3.81	4.5	115.54 ± 6.71	5.8
	200	93.58 ± 4.14	4.4	103.39 ± 7.55	7.3
baohuoside I	2	99.45 ± 6.74	6.8	93.08 ± 5.89	6.3
	20	97.78 ± 1.36	1.4	103.85 ± 6.55	6.3
	400	98.50 ± 1.73	1.8	98.21 ± 3.20	3.3
sagittatoside A	2	74.52 ± 7.02	9.4	112.96 ± 8.54	7.6
	20	79.30 ± 3.64	4.6	118.72 ± 9.92	8.4
	400	90.06 ± 3.35	3.7	107.33 ± 10.01	9.3
sagittatoside B	2	99.77 ± 4.19	4.2	98.77 ± 3.97	4.0
	20	82.34 ± 4.82	5.9	112.35 ± 7.63	6.8
	400	86.73 ± 2.69	3.1	111.49 ± 6.28	5.6
epimedin A	2	60.66 ± 6.63	10.9	113.02 ± 10.87	9.6
	20	68.52 ± 0.66	1.0	119.11 ± 13.22	11.1
	400	84.85 ± 3.63	4.3	112.83 ± 9.14	8.1
epimedin B	2	70.66 ± 4.43	6.3	119.84 ± 6.79	5.7
	20	94.72 ± 0.75	0.8	107.49 ± 8.41	7.8
	400	75.30 ± 1.12	1.5	119.30 ± 3.05	2.6
epimedin C	2	96.35 ± 1.00	1.0	102.59 ± 3.00	2.9
	20	70.91 ± 2.30	3.2	112.31 ± 11.51	10.2
	400	81.57 ± 1.92	2.4	118.74 ± 6.52	5.5
magnoflorine	4	61.95 ± 6.86	11.1	115.27 ± 15.25	13.2
	40	70.29 ± 4.68	6.7	114.36 ± 5.95	5.2
	800	81.33 ± 4.59	5.6	110.84 ± 12.78	11.5
chlorogenic acid	10	97.79 ± 4.36	4.5	98.55 ± 7.49	7.6
	100	89.78 ± 8.65	9.6	98.36 ± 5.32	5.4
	2000	78.81 ± 5.22	6.6	114.50 ± 13.84	12.1
neochlorogenic acid	4	82.92 ± 2.58	3.1	113.90 ± 5.98	5.3
	40	78.53 ± 2.88	3.7	114.36 ± 5.95	5.2
	800	84.04 ± 3.36	4.0	115.44 ± 8.99	7.8
cryptochlorogenic acid	4	91.38 ± 10.31	11.3	96.33 ± 6.89	7.2
	40	76.05 ± 6.75	8.9	118.92 ± 11.70	9.8
	800	76.95 ± 3.26	4.2	116.51 ± 12.06	10.4
icariin	4	96.32 ± 4.65	4.8	95.68 ± 5.32	5.6
	40	88.84 ± 9.11	10.3	111.87 ± 10.96	9.8
	800	91.78 ± 4.42	4.8	105.94 ± 8.98	8.5

**Table 4:** Stability of all analytes in rat plasma (*n* = 3).

Compounds	Spiked Concentration (ng/mL)	Room Temperature for 2 h		Three Freeze-Thaw Cycles		Auto-Sampler for 12 h		−70 °C for 7 Days	
		Measured (ng/mL)	RSD (%)	Measured (ng/mL)	RSD (%)	Measured (ng/mL)	RSD (%)	Measured (ng/mL)	RSD (%)
baohuoside II	1	0.96 ± 0.09	9.4	1.09 ± 0.02	1.8	1.10 ± 0.11	10.0	1.04 ± 0.12	11.5
	10	9.99 ± 0.31	3.1	8.94 ± 0.63	7.0	10.38 ± 0.60	5.8	8.84 ± 0.31	3.5
	200	193.54 ± 3.74	1.9	195.26 ± 1.32	0.7	190.62 ± 5.99	3.1	188.79 ± 7.35	3.9
baohuoside I	2	1.93 ± 0.03	1.6	1.97 ± 0.08	4.1	2.01 ± 0.07	3.5	2.03 ± 0.09	4.4
	20	19.56 ± 0.23	1.2	19.71 ± 0.49	2.5	19.24 ± 0.96	5.0	19.48 ± 0.52	2.7
	400	389.39 ± 7.29	1.9	393.71 ± 5.02	1.3	387.09 ± 9.42	2.4	391.78 ± 3.49	0.9
sagittatoside A	2	2.08 ± 0.12	5.8	2.11 ± 0.07	3.3	1.93 ± 0.09	4.7	1.98 ± 0.12	6.1
	20	20.97 ± 0.09	0.4	19.84 ± 0.81	4.1	21.29 ± 0.18	0.8	19.43 ± 0.40	2.1
	400	397.27 ± 8.71	2.2	393.69 ± 3.71	0.9	397.42 ± 6.71	1.7	395.24 ± 11.34	2.9
sagittatoside B	2	2.04 ± 0.16	7.8	2.09 ± 0.05	2.4	2.07 ± 0.08	3.9	2.03 ± 0.14	6.9
	20	21.32 ± 0.59	2.8	20.07 ± 0.95	4.7	21.16 ± 0.51	2.4	18.84 ± 0.52	2.8
	400	398.03 ± 9.83	2.5	394.07 ± 3.55	0.9	407.64 ± 17.60	4.3	381.68 ± 5.87	1.5
epimedin A	2	2.10 ± 0.09	4.3	2.07 ± 0.13	6.3	2.06 ± 0.04	1.9	2.03 ± 0.09	4.4
	20	20.29 ± 0.43	2.1	19.58 ± 0.94	4.8	20.35 ± 0.78	3.8	19.70 ± 1.54	7.8
	400	400.51 ± 0.52	0.1	392.00 ± 2.85	0.7	398.73 ± 3.31	0.8	400.95 ± 4.79	1.2
epimedin B	2	2.01 ± 0.04	2.0	2.07 ± 0.09	4.3	2.18 ± 0.05	2.3	2.07 ± 0.09	4.3
	20	21.51 ± 0.97	4.5	20.27 ± 1.82	9.0	21.01 ± 0.50	2.4	21.00 ± 0.78	3.7
	400	400.89 ± 3.17	0.8	400.51 ± 2.96	0.7	397.07 ± 2.81	0.7	403.31 ± 2.82	0.7
epimedin C	2	2.10 ± 0.05	2.4	2.03 ± 0.10	4.9	2.02 ± 0.14	6.9	2.14 ± 0.05	2.3
	20	20.24 ± 0.83	4.1	20.43 ± 0.90	4.4	19.73 ± 0.37	1.9	21.37 ± 0.10	0.5
	400	399.97 ± 1.97	0.5	397.64 ± 8.12	2.0	396.56 ± 4.69	1.2	396.46 ± 8.82	2.2
magnoflorine	4	3.99 ± 0.08	2.0	4.01 ± 0.13	3.2	4.37 ± 0.19	4.3	4.02 ± 0.10	2.5
	40	39.29 ± 2.92	7.4	41.99 ± 2.30	5.5	41.70 ± 1.06	2.5	40.27 ± 1.70	4.2
	800	776.84 ± 1.85	0.2	780.80 ± 15.38	2.0	809.27 ± 12.34	1.5	775.71 ± 3.82	0.5
chlorogenic acid	10	9.93 ± 0.03	0.3	9.52 ± 0.84	8.8	9.26 ± 0.68	7.3	8.88 ± 0.39	4.4
	100	101.51 ± 11.83	11.7	91.99 ± 6.26	6.8	98.92 ± 4.58	4.6	85.73 ± 4.01	4.7
	2000	1866.65 ± 25.87	1.4	1883.55 ± 25.34	1.3	1924.54 ± 82.95	4.3	1849.43 ± 30.88	1.7
neochlorogenic acid	4	4.00 ± 0.14	3.5	3.97 ± 0.05	1.3	3.85 ± 0.14	3.6	4.13 ± 0.06	1.5
	40	39.79 ± 2.13	5.4	38.73 ± 1.14	2.9	41.77 ± 1.37	3.3	39.14 ± 1.82	4.6
	800	802.82 ± 7.13	0.9	791.74 ± 0.87	0.1	803.36 ± 2.53	0.3	804.80 ± 4.45	0.6
cryptochlorogenic acid	4	4.00 ± 0.14	3.5	4.03 ± 0.13	3.2	4.01 ± 0.22	5.5	4.01 ± 0.06	1.5
	40	40.59 ± 1.78	4.4	39.42 ± 1.80	4.6	41.58 ± 0.51	1.2	39.06 ± 1.65	4.2
	800	773.95 ± 5.78	0.7	781.42 ± 2.45	0.3	768.52 ± 23.65	3.1	759.75 ± 7.73	1.0
icariin	4	4.07 ± 0.10	2.5	4.09 ± 0.09	2.2	4.11 ± 0.10	2.4	4.00 ± 0.06	1.5
	40	40.07 ± 0.42	1.0	40.45 ± 1.98	4.9	40.34 ± 1.48	3.7	40.22 ± 0.40	1.0
	800	799.61 ± 7.41	0.9	796.41 ± 5.25	0.7	808.06 ± 2.49	0.3	801.34 ± 5.26	0.7

**Table 5:** Pharmacokinetic parameters of 12 analytes after oral administration of *Epimedium* extract (*n* = 6).

Compounds	*T_max1_* (h)	*T_max2_* (h)	*C_max1_* (ng/mL)	*C_max2_* (ng/mL)	*t_1/2_* (h)	*AUC_(0-tn)_* (h·ng/mL)	*AUC_(0-∞)_* (h·ng/mL)	*MRT_(0-tn)_* (h)	*MRT_(0-∞)_* (h)
baohuoside II	0.18 ± 0.03	10.67 ± 1.03	6.87 ± 4.58	29.17 ± 7.70	6.93 ± 2.94	245.95 ± 133.39	354.89 ± 143.76	11.47 ± 1.99	16.50 ± 3.06
baohuoside I	0.38 ± 0.14	10.00 ± 1.79	65.20 ± 33.56	170.73 ± 66.97	13.25 ± 4.80	1448.99 ± 615.56	1675.75 ± 598.59	10.81 ± 1.21	15.68 ± 4.10
sagittatoside A	0.18 ± 0.03	11.67 ± 0.82	20.35 ± 10.26	203.50 ± 97.03	13.81 ± 7.09	1614.13 ± 348.72	1782.28 ± 351.94	12.02 ± 0.71	14.26 ± 2.00
sagittatoside B	0.20 ± 0.04	11.00 ± 1.67	19.38 ± 9.79	112.97 ± 44.50	9.38 ± 2.11	853.80 ± 171.13	1036.71 ± 201.70	11.67 ± 1.17	14.89 ± 2.09
epimedin A	0.22 ± 0.04	9.33 ± 0.94	82.79 ± 7.61	59.34 ± 26.24	12.50 ± 6.85	550.27 ± 199.35	600.14 ± 218.86	10.82 ± 2.33	14.06 ± 3.98
epimedin B	0.19 ± 0.07	7.67 ± 0.75	316.73 ± 59.06	129.90 ± 77.04	3.64 ± 3.68	945.89 ± 250.22	1066.50 ± 212.44	6.98 ± 1.14	8.37 ± 2.39
epimedin C	0.22 ± 0.04	7.67 ± 0.75	135.22 ± 31.26	103.20 ± 33.10	17.85 ± 8.23	850.40 ± 268.63	919.35 ± 214.91	9.33 ± 2.34	12.54 ± 2.59
magnoflorine	0.18 ± 0.03	10.67 ± 1.63	411.40 ± 110.77	275.67 ± 89.36	17.77 ± 8.25	3676.19 ± 874.51	4003.32 ± 969.59	9.86 ± 0.75	12.52 ± 1.72
chlorogenic acid	0.19 ± 0.05		540.28 ± 225.18		0.45 ± 0.30	879.68 ± 365.08	886.48 ± 376.98	3.85 ± 1.28	3.91 ± 1.32
neochlorogenic acid	0.19 ± 0.05		205.96 ± 75.12		0.64 ± 0.27	263.82 ± 68.24	268.58 ± 70.86	3.18 ± 1.57	3.25 ± 1.97
cryptochlorogenic acid	0.19 ± 0.05		269.77 ± 114.07		0.53 ± 0.24	397.96 ± 170.58	404.11 ± 168.92	3.75 ± 1.91	3.82 ± 2.48
icariin	0.16 ± 0.05		350.28 ± 195.96		0.41 ± 0.19	322.75 ± 204.68	324.78 ± 209.27	2.71 ± 0.79	2.75 ± 0.97

**Table 6:** Mass spectra properties of 12 analytes and IS.

Compounds	Precursor Ion (*m*/*z*)	Product Ion (*m*/*z*)	Frag. (V)	C.E. (V)
baohuoside II	499.2	353.0	140	20
baohuoside I	513.2	366.0	140	20
sagittatoside A	675.2	367.0	145	32
sagittatoside B	645.2	366.2	145	30
epimedin A	675.1	365.8	130	32
epimedin B	645.1	365.6	145	30
epimedin C	659.2	365.7	145	30
magnoflorine	340.1	310.1	145	22
chlorogenic acid	353.0	191.0	90	10
neochlorogenic acid	352.9	191.0	115	10
cryptochlorogenic acid	353.1	172.9	100	10
icariin	721.0	513.2	145	10
liquiritin (IS)	417.1	255.0	145	13
